# Leukocyte cell population data predict 30-day mortality in community-acquired pneumonia: A multicenter prospective study

**DOI:** 10.17305/bb.2026.14362

**Published:** 2026-06-24

**Authors:** Ane Uranga, Eloísa Urrechaga, Urko Aguirre, Amaia Artaraz, Leire Davila, Maider Intxausti, Cristina Sancho, Carlos Ruiz-Martinez, Javier Ugedo, Cristina Ponga, Jose María Quintana, Pedro Pablo España, and the LEUCOCAP Study Group

**Affiliations:** 1 Department of Pneumology, Biobizkaia Health Research Institute, Osakidetza Basque Health Service, Galdakao-Usansolo University Hospital, Galdakao, Bizkaia, Spain; 2 Clinical Laboratory, Biobizkaia Health Research Institute, Osakidetza Basque Health Service, Galdakao-Usansolo University Hospital, Galdakao, Bizkaia, Spain; 3 Research Unit, Galdakao-Usansolo University Hospital, Osakidetza Basque Health Service, Galdakao, Spain; 4 Health Service Research Network on Chronic Diseases (REDISSEC), Bizkaia, Spain; 5 Biosistemak, Institute for Health Services Research, Bilbao, Spain; 6 Network for Research on Chronicity, Primary Care, and Health Promotion (RICAPPS), Galdakao, Spain; 7 Department of Pneumology, University Hospital of Alava, Vitoria-Gasteiz, Spain; 8 Department of Pneumology, University Hospital of Basurto, Bilbao, Spain; 9 Department of Pneumology, University Hospital of San Pedro, Logroño, La Rioja, Spain

**Keywords:** Leukocyte, cell population data, pneumonia, Sysmex XN, mortality.

## Abstract

Early identification of patients with community-acquired pneumonia (CAP) at increased risk of death remains essential, but rapid and low-cost prognostic biomarkers are still needed. Cell population data (CPD), automatically generated during routine complete blood count (CBC) testing, reflect leukocyte morphology and functional activation. This study aimed to assess whether CPD parameters predict 30-day mortality in hospitalized patients with CAP. This multicenter, observational, prospective study included consecutive adults admitted with CAP to three teaching hospitals in northern Spain between November 2019 and November 2021. A Sysmex XN-20 analyzer was used to measure leukocyte CPD parameters at baseline and at Day 3–5. Prediction models for 30-day all-cause mortality were developed using baseline CPD values and, in the follow-up cohort, early changes in CPD values. A total of 1,782 patients were included, of whom 1,480 had Day 3–5 follow-up data; 98 patients died within 30 days. In the baseline cohort, coronavirus disease 2019 (COVID-19) status, male sex, age ≥65 years, neutrophil fluorescence distribution width (NE-WY) ≥617.4, and monocyte fluorescence intensity (MO-Y) ≥130 predicted 30-day mortality, with an area under the receiver operating characteristic curve (AUC) of 0.81 [95% confidence interval (CI): 0.76–0.85]. In the follow-up cohort, COVID-19 status, age ≥65 years, male sex, baseline neutrophil fluorescence intensity (NE-Y) ≥51.63, MO-Y ≥130, NE-Y increase ≥3.76, and MO-Y decrease ≤–12.49 predicted mortality, with an AUC of 0.82 (95% CI: 0.76–0.88). CPD-based leukocyte parameters may provide a rapid, accessible, and cost-effective tool for identifying hospitalized patients with CAP at increased risk of 30-day mortality.

## Introduction

Community-acquired pneumonia (CAP) presents a significant burden on public healthcare systems, characterized by high morbidity and mortality rates [1]. To stratify the risk of mortality in CAP patients, numerous prognostic scoring systems have been developed [2, 3]. While inflammatory biomarkers such as C-reactive protein (CRP) have demonstrated utility in identifying adverse outcomes [4], their predictive ability for mortality risk remains lower than that of clinical scores. Other biomarkers, including procalcitonin (PCT) and proadrenomedullin, exhibit improved predictive accuracy for severe outcomes; however, their widespread clinical implementation is hindered by reduced diagnostic sensitivity, high cost, and practical challenges [4–6].

Recent technological advancements have led to rapid developments in hematology analyzers, enabling the reporting of novel parameters beyond the standard complete blood count (CBC). Cell population data (CPD) are background parameters that provide quantitative insights into the morphological and functional characteristics of blood cells, specifically describing features such as granularity, volume, and nucleic acid/protein content.

Abnormalities in white blood cell morphology (size, shape, and composition) have been linked to infection response [7, 8]. These abnormalities are hypothesized to reflect diverse pathogenic causes of infection and varying early innate, cellular, or humoral immune responses [9]. Changes in CPD parameters, such as alterations in neutrophil and monocyte volumes, have been identified as early manifestations of immune cell response to sepsis. Consequently, the Neutrophil and Monocyte score (NEMO), which incorporates various CPD parameters, has been developed to detect the probability of sepsis with high predictive accuracy [10].

During the 2019 SARS-CoV-2 pandemic, associations were observed between COVID-19 infection and abnormalities in leukocyte count morphology, as well as lymphopenia. Several studies have highlighted a close association between CPD and SARS-CoV-2 infection [11–13]. Furthermore, CPD parameters have proven useful in differentiating between pneumonia caused by COVID-19 and pneumonia resulting from other etiologies [14].

Despite their utility in detecting SARS-CoV-2 infection, the specific role of these parameters in identifying CAP patients who will develop severe outcomes remains largely unexplored. Buoro et al. reported a significant correlation between certain CPD parameters and the Sequential Organ Failure Assessment (SOFA) score in septic patients, suggesting their potential as a valuable tool for sepsis management in the intensive care unit (ICU) [15]. A recent study also demonstrated the utility of CPD, obtained from complete blood analysis, and free light chain (FLC) concentration in distinguishing between severe and non-severe COVID-19 [16].

Accurate initial assessment of disease severity is crucial for identifying patients at risk of complications and facilitating intensified monitoring. CPD parameters, reflecting white blood cell characteristics and morphological variations, may serve as a valuable prognostic tool to improve CAP patient management. Therefore, this study aimed to evaluate the ability of CPD parameters to predict mortality in patients hospitalized with CAP.

## Materials and methods

### Setting and study population

This multicenter prospective observational study enrolled consecutive hospitalized CAP patients from November 2019 to November 2021 across three hospitals in northern Spain. The study received approval from the Regional Ethics Committee (PI2019090, 17 July 2019) and was registered on ClinicalTrials.gov (NCT 04930926). All participants provided informed consent after receiving comprehensive information about the study’s objectives, risks, and potential benefits.

Eligible patients were aged ≥18 years and hospitalized with a CAP diagnosis. Pneumonia was defined by the presence of a new pulmonary infiltrate on chest X-ray coupled with at least one symptom consistent with pneumonia (e.g., cough, fever, dyspnea, and/or chest pain) [17]. Exclusion criteria included discharge from an acute care hospital, onsite subacute care unit, or palliative care unit within the preceding 14 days; human immunodeficiency virus (HIV) positivity; or chronic immunosuppression (defined as immunosuppression for solid organ transplantation, splenectomy, prednisone use ≥10 mg/d or equivalent for >30 days, use of other immunosuppressive agents, or neutropenia, i.e., neutrophil count <109/L).

### Data collection

Demographic and baseline clinical data, including comorbidities, physical examination findings, radiological presentation, laboratory test results, and complications during admission, were extracted from patient medical records. Disease severity was assessed within 24 h of diagnosis using the Confusion, Urea, Respiratory rate, Blood pressure, and age >65 (CURB-65) score, Pneumonia Severity Index (PSI) score, and severe community-acquired pneumonia score (SCAP) [2].

At baseline, a Sysmex XN-20 analyzer was used to obtain detailed information on the morphological and functional characteristics of white blood cells. This included internal cellular complexity (NE-X, LY-X, MO-X, NE-WX, LY-WX, MO-WX), cell size (NE-Z, LY-Z, MO-Z, NE-WZ, LY-WZ, MO-WZ), and nucleic acid quantity (NE-Y, LY-Y, MO-Y, NE-WY, LY-W, MO-WY). Differences in optical signals, arising from variations in membrane composition between activated and resting cells, enable the quantification of morphological changes directly related to cellular functionality through numerical CPD values. Samples, collected in K2 EDTA anti-coagulant tubes (VacutainerTM Becton-Dickinson, Rutherford, NJ, USA) as part of routine analysis, were processed on the Sysmex XN-20 analyzer within 4 h of collection, adhering to standard laboratory procedures. All participating centers utilized identical Sysmex hematology analyzer platforms and maintained routine internal and external quality-control procedures in accordance with institutional protocols and manufacturer recommendations.

### Assessment of outcomes

The primary outcome was all-cause mortality within 30 days of hospital admission for CAP. The secondary outcome was 30-day mortality among patients from whom a blood sample was obtained at Day 3–5, to assess the impact of changes in CPD parameters on mortality. Vital status at 30 days was determined through electronic medical record review. Patients were followed for the entire 30-day period, with no loss to follow-up.

### Statistical analysis

Continuous variables were expressed as means (or medians) and standard deviations (or interquartile range), while categorical variables were presented as frequencies and percentages.

The study sample was randomly divided into derivation (training, 60%) and test (40%) datasets. Baseline differences between these datasets were compared using the non-parametric Wilcoxon and chi-square tests.

Two scores were developed and validated for predicting 30-day mortality: a) using baseline CPD and inflammatory markers retrieved upon Emergency Department admission; and b) incorporating measurements of the aforementioned variables at both baseline and Day 3 or 5. The entire eligible cohort was utilized for the first objective, while only patients with Day 3–5 follow-up data (cohort with follow-up) were included for the second objective.

Score development involved the following steps: first, variables between the training and test sets were compared using the non-parametric Wilcoxon, chi-square, and Fisher’s Exact tests.

Within the training set, the chi-square or Fisher’s Exact test and the Wilcoxon test were employed to assess the relationship between categorical and continuous variables and 30-day mortality. Continuous variables were then optimally categorized using an algorithm (CatPredi package). Variables with a *P-*value <0.20 were subsequently included in a multivariable logistic regression model. Final independent predictors were identified using a backward selection procedure. A score was assigned to each covariate within the nomogram’s components by delineating a corresponding vertical line intersecting the labeled axis elements. The individual probabilities of 30-day mortality generalization were ascertained by summing the total score and locating it on the total points scale. Risk groups were subsequently determined in the test set.

We assessed model robustness by evaluating discrimination and calibration in two distinct datasets. Discrimination was quantified using the area under the ROC curve (AUC). In the training dataset, both the apparent AUC and the optimism-corrected AUC were estimated via bootstrap resampling (*B* ═ 2,000 samples) to mitigate potential overfitting from variable selection. For the test dataset, the apparent AUC was reported with 95% confidence intervals, derived from 2,000 bootstrap samples, to ensure robust and precise estimates of model performance. An AUC exceeding 0.80 was considered indicative of strong model discrimination. Calibration was graphically assessed through calibration curves (estimating intercepts and slopes) and by calculating the Brier score. A Brier score closer to 0 indicates superior calibration. For calibration intercept-slopes, a slope near 1.0 and an intercept near 0 signify excellent alignment between predicted risks and observed outcomes.

Clinical utility was evaluated using decision curve analysis (DCA) across a range of threshold probabilities, with a primary focus on the 5%–10% range. This range was selected based on the observed 30-day mortality (4.5%) and established estimates for CAP. Both continuous probabilities and categorical risk groups were incorporated into the analyses. The incremental value of the CPD score over CURB-65 was determined using Net Reclassification Improvement (NRI) and Integrated Discrimination Improvement (IDI), employing both continuous and categorical approaches. *A priori* risk categories were defined as low (<5%), intermediate (5%–15%), and high (≥15%). Given the limited number of events (*n* ═ 32), categorical NRI was prioritized as the primary metric due to the potential instability and reduced clinical interpretability of continuous measures in such cohorts. Analyses were conducted using PredictABEL and nricens packages, with 1,000 bootstrap iterations and a significance level of α ═ 0.05.

All statistical analyses were performed using SAS software v9.4 and the R statistical package (version 4.0.4, R Foundation for Statistical Computing). A *P-*value less than 0.05 was considered statistically significant.

## Results

### Descriptive statistics of the study cohorts

A total of 1,782 patients from three academic hospitals were included in the analysis. Of these, 1,480 (83.05%) had follow-up data available at Day 3–5. The study flow chart is presented in Figure 1. SARS-CoV-2 infection was confirmed in 71.36% of patients. No significant differences were observed in baseline sociodemographic and clinical characteristics between patients with and without Day 3–5 follow-up (Table S1). Within the CPD data, significant differences were noted for NE-WY, NE-Z, MO-X, CRP, and PCT, all of which were higher in patients with Day 3–5 follow-up. Conversely, platelet-to-lymphocyte ratio (PLR) values were higher in patients without follow-up. The mean age (SD) of the total cohort was 62.7 (15.1) years, and 1,158 (65%) patients were male. Furthermore, 37.9% of patients had a Charlson Comorbidity Index (CCI) score of ≥1 point, and 71.5% tested positive for SARS-CoV-2 infection. Thirty-day mortality occurred in 98 (5.5%) patients within the total cohort.

**Figure 1.**
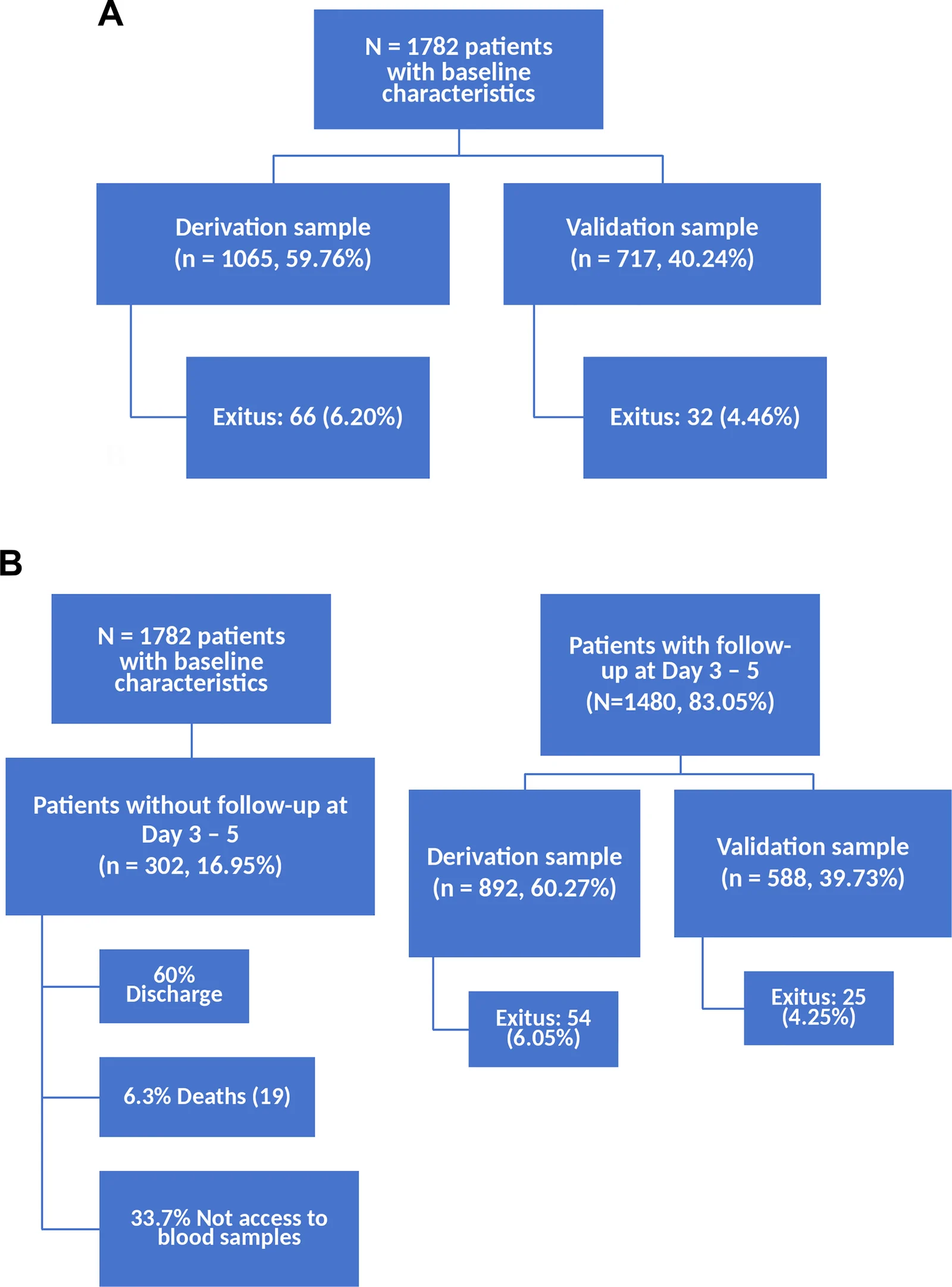
**Flowchart for predicting mortality using baseline characteristics (A) and combined baseline and follow-up characteristics (B).** **Abbreviation:** n, number of patients.

**Table 1:** Unadjusted associations between patient characteristics and 30-day mortality in the training sample

	**30-day status**		
**Variables**	**Alive (*n* ═ 999)**	**Dead (*n* ═ 66)**	**Total (*n* ═ 1065)**	***P* value**
**Gender**
Male	627 (92.61)	50 (7.39)	677 (63.6)	0.03
Female	372 (95.9)	16 (4.1)	388 (36.4)	
**Age, mean (SD)**	62.2 (14.9)	76.0 (11.7)	63.1 (15.1)	<.0001
**Living in residential care**	13 (59.1)	9 (40.9)	22 (2.1)	<.0001
**Charlson Comorbidity Index**				0.0022
0	630 (95.60)	29 (4.40)	659 (61.9)	
1	206 (92.79)	16 (7.21)	222 (20.8)	
2	82 (91.11)	8 (9.89)	90 (8.5)	
3	81 (86.17)	13 (13.83)	94 (8.8)	
**SARS-CoV-2 infection:** **Positive**	707 (93.02)	53 (6.97)	760 (71.4)	0.10
Cell Population Data^*^				
NE-X	155.00 (151.00, 158.0)	154.70 (151.00, 158.0)	155.00 (151.00, 158.0)	0.84
NE-WX	308.00 (298.00, 321.0)	312.50 (302.00, 326.0)	308.00 (298.00, 321.0)	0.15
NE-Y	51.00 (49.00, 54.0)	51.00 (48.00, 54.0)	51.00 (49.00, 54.0)	0.84
NE-WY	613.00 (583.00, 657.0)	631.50 (608.00, 673.0)	614.00 (583.00, 659.0)	0.007
NE-Z	87.50 (84.40, 90.6)	87.15 (85.10, 90.5)	87.50 (84.50, 90.6)	0.94
NE-WZ	660.00 (627.00, 709.0)	666.00 (633.00, 725.0)	661.00 (627.00, 709.0)	0.400
LY-X	83.30 (81.40, 84.8)	83.70 (81.40, 85.1)	83.30 (81.40, 84.8)	0.27
LY-WX	489.00 (449.00, 536.0)	471.50 (427.00, 517.0)	488.00 (447.00, 534.0)	0.02
LY-Y	72.20 (68.90, 75.1)	71.35 (69.20, 75.6)	72.10 (68.90, 75.1)	0.80
LY-WY	808.00 (737.00, 885.0)	776.50 (713.00, 863.0)	806.00 (735.00, 885.0)	0.10
LY-Z	61.00 (59.10, 62.6)	61.40 (59.10, 63.3)	61.00 (59.10, 62.6)	0.24
LY-WZ	537.00 (495.00, 577.0)	514.50 (478.00, 574.0)	536.00 (493.00, 577.0)	0.16
MO-X	125.00 (123.70, 127.6)	126.00 (123.00, 128.0)	125.00 (123.50, 127.8)	0.58
MO-WX	241.00 (226.00, 262.0)	248.50 (225.00, 275.0)	242.00 (226.00, 262.0)	0.19
MO-Y	119.00 (112.90, 126.0)	121.50 (113.00, 131.0)	119.00 (113.00, 126.0)	0.11
MO-WY	671.00 (592.00, 739.0)	617.50 (547.00, 700.0)	669.00 (588.00, 739.0)	0.01
MO-Z	66.80 (64.30, 69.4)	67.75 (64.40, 70.1)	66.80 (64.30, 69.5)	0.22
MO-WZ	599.00 (553.00, 661.0)	588.00 (527.00, 635.0)	598.00 (552.00, 660.0)	0.10
Inflammatory markers^*^				
CRP	78.00 (40.10, 136.0)	89.25 (41.60, 158.0)	79.10 (40.10, 137.0)	0.21
PCT	0.11 (0.07, 0.3)	0.17 (0.10, 0.5)	0.12 (0.07, 0.3)	0.001
NLR	6.16 (3.59, 11.2)	8.03 (5.09, 14.3)		0.01
PLR	155.43 (43.51, 251.8)	154.19 (38.93, 320.0)		0.52

^*^Data shown as median (25^th^–75^th^ percentiles). Categorical data are presented as n (%). **Abbreviations**: CRP, C-reactive protein; LY-WX, lymphocyte internal-complexity distribution width; LY-WY, lymphocyte fluorescence distribution width; LY-WZ, lymphocyte cell-size distribution width; LY-X, lymphocyte internal complexity; LY-Y, lymphocyte fluorescence intensity; LY-Z, lymphocyte cell size; MO-WX, monocyte internal-complexity distribution width; MO-WY, monocyte fluorescence distribution width; MO-WZ, monocyte cell-size distribution width; MO-X, monocyte internal complexity; MO-Y, monocyte fluorescence intensity; MO-Z, monocyte cell size; NE-WX, neutrophil internal-complexity distribution width; NE-WY, neutrophil fluorescence distribution width; NE-WZ, neutrophil cell-size distribution width; NE-X, neutrophil internal complexity; NE-Y, neutrophil fluorescence intensity; NE-Z, neutrophil cell size; NLR, neutrophil-to-lymphocyte ratio; PCT, procalcitonin; PLR, platelet-to-lymphocyte ratio; SARS-CoV-2, severe acute respiratory syndrome coronavirus 2; SD, standard deviation.

### Mortality prediction in the baseline cohort

Table 1 and Table S2 present the baseline characteristics of 30-day survivors and non-survivors in the training sample. Non-survivors exhibited significantly higher PCT levels. Predictors of 30-day mortality included positive COVID status, male gender, age ≥ 65 years, NE-WY ≥ 617.4, and MO-Y ≥ 130 (Table 2). A prediction score for 30-day mortality was developed in the validation sample, assigning 7 points for age ≥ 65 years and 2 points each for male gender, positive COVID status, NE-WY ≥ 617, and MO-Y ≥ 130. This score demonstrated high predictive accuracy (AUC 0.81; 95% CI: 0.76,0.85). The risk score was subsequently categorized into three groups, with the highest risk observed in patients scoring ≥15 points (AUC 0.77; 95% CI: 0.69,0.82) (Table 3). Figure 2 displays the ROC curves for the scores in both the training and test samples. A sub-analysis incorporating PCT into the model revealed that PCT did not achieve statistical significance after adjustment for age, gender, and COVID status (*P ═* 0.15). Calibration was excellent in the derivation cohort (intercept = 0, slope = 1.0, Brier score = 0.04). In the validation cohort, the intercept (0.059) and slope (1.164) showed minor deviations from ideal values, suggesting a slight overestimation at higher predicted probabilities; however, overall calibration remained acceptable (Brier score = 0.041). In the validation cohort, the continuous CPD score achieved an AUC of 0.80 (95% CI: 0.75–0.85), which was comparable to CURB-65 (AUC 0.84, *P ═* 0.168) and PSI (AUC 0.80, *P ═* 0.638), and numerically superior to SCAP (AUC 0.67, *P ═* 0.211), CRP (AUC 0.74, *P ═* 0.068), and PCT (AUC 0.74, *P ═* 0.075). In the categorical analysis, the CPD score (AUC 0.76, 95% CI: 0.69–0.83) significantly outperformed categorical CURB-65 (AUC 0.62, *P ═* 0.008) and PSI (AUC 0.56, *p* < 0.001). No significant differences were observed when compared against categorical SCAP (*P ═* 0.218), CRP (*P ═* 0.381), or PCT (*P ═* 0.193) (Table S3).

**Table 2:** Multivariate logistic regression analysis of the association between baseline CPD measurement and 30-day mortality in training samples from the baseline cohort (*n* ═ 1065) and the follow-up cohort (*n* ═ 892)

**Variable**	**Beta (s.e.)**	**OR (95% CI)**	***P* value**	**Weight**
**Baseline cohort (*n* = 1065)**				
NE-WY≥ 617.4	0.72 (0.29)	2.05 (1.17, 3.60)	0.01	2
MO-Y≥ 130	0.70 (0.28)	2.02 (1.17, 3.48)	0.01	2
Gender: Male	0.69 (0.31)	2.00 (1.10, 3.63)	0.02	2
Age ≥ 65 years	2.31 (0.39)	10.05 (4.69, 21.52)	<0.001	7
Status COVID: Positive	0.78 (0.35)	2.18 (1.10, 4.35)	0.03	2
AUC (95% CI)^*^	0.81 (0.76, 0.85)			
**Cohort with follow-up (*n* = 892)**				
Age ≥ 65 years	2.09 (0.43)	8.09 (3.48, 18.83)	<0.001	5
Gender: Male	0.85 (0.40)	2.33 (1.07, 5.08)	0.03	2
Baseline NE-Y≥ 51.63	0.48 (0.34)	1.62 (0.83, 3.16)	0.16	0
Baseline MO-Y≥ 130	0.45 (0.42)	1.57 (0.68, 3.60)	0.29	0
Change from baseline to Day 3–5 in:				
NE-Y≥3.76	1.74 (0.59)	5.71 (1.80, 18.08)	0.003	4
MO-Y≤ -12.49	0.86 (0.40)	2.36 (1.07, 5.20)	0.03	2
Status COVID: Positive	1.25 (0.55)	3.49 (1.19, 10.21)	0.02	3
				
AUC (95% CI)^*^	0.82 (0.76, 0.88)			
				

*After bootstrap resampling (2000 samples), we calculated the beta regression coefficient (standard error). Zero-weight variables (baseline NE-Y ≥51.63 and MO-Y ≥130) were retained as adjusting covariates to account for baseline values when modeling their respective Day 3–5 changes. Their non-zero regression coefficients did not meet the threshold for a non-zero integer score point under the rounding procedure applied during score construction. **Abbreviations**: AUC, area under the receiver operating characteristic curve; CI, confidence interval; COVID-19, coronavirus disease 2019; CPD, cell population data; MO-Y, monocyte fluorescence intensity; n, number of patients; NE-WY, neutrophil fluorescence distribution width; NE-Y, neutrophil fluorescence intensity; OR, odds ratio; ROC, receiver operating characteristic; s.e., standard error.

**Table 3:** Risk-group logistic regression analysis of 30-day mortality based on established risk levels within the score, applied to baseline (*n* ═ 717) and follow-up (*n* ═ 588) test cohorts

	**Event/n patients**	**% Events (95% CI)**	**OR (95% CI)**	***P* value**
**Baseline cohort (*n* = 717)**				
Continuous score	-		1.02 (1.01, 1.02)	<0.001
AUC (95% CI)			0.80 (0.75, 0.85)	
AUC (95% CI)*			0.80 (0.74, 0.85)	
Brier score			0.04	
Risk groups				
≤6	1/364	0.3 (0.007, 1.521)	Reference	
7-14	26/324	8 (5.309, 11.537)	1.06 (1.03, 1.10)	<0.001
≥15	5/29	17.24 (5.845, 35.775)	1.11 (1.07, 1.15)	<0.001
AUC (95% CI)			0.76 (0.69, 0.83)	
AUC (95% CI)^**^			0.77 (0.69, 0.82)	
Brier score			0.04	
**Cohort with follow-up (*n* = 588)**				
Score continuous	-		1.44 (1.21, 1.71)	<0.001
AUC (95% CI)			0.79 (0.71, 0.87)	
AUC (95% CI)*			0.80 (0.71, 0.87)	
Brier score			0.035	
Risk groups				
≤7	4/318	1.30 (0.344, 3.189)	Reference	
8-10	10/157	6.37 (3.096, 11.401)	5.34 (1.65, 17.31)	<0.001
≥11	5/28	17.90 (0.566, 36.893)	17.07 (4.29, 67.92)	<0.001
AUC (95% CI)			0.76 (0.64, 0.87)	
AUC (95% CI)^**^			0.76 (0.64, 0.87)	
Brier score			0.034	

*After bootstrapping with 2,000 samples, 95% confidence intervals (95% CI) were calculated. OR, odds ratio; AUC, area under the receiver operating characteristic (ROC) curve calculated without bootstrapping. Event/N patients indicates the number of patients who experienced the event of interest out of the total number of patients in the corresponding group. **In the follow-up validation cohort, 25 deaths occurred among 588 patients. Risk groups could be assigned to 503 patients, including 19 deaths. The remaining 85 patients were excluded because of missing values in at least one score component; no imputation was performed. **Abbreviations**: AUC, area under the receiver operating characteristic curve; CI, confidence interval; CPD, cell population data; n, number of patients; OR, odds ratio; ROC, receiver operating characteristic.

**Figure 2.**
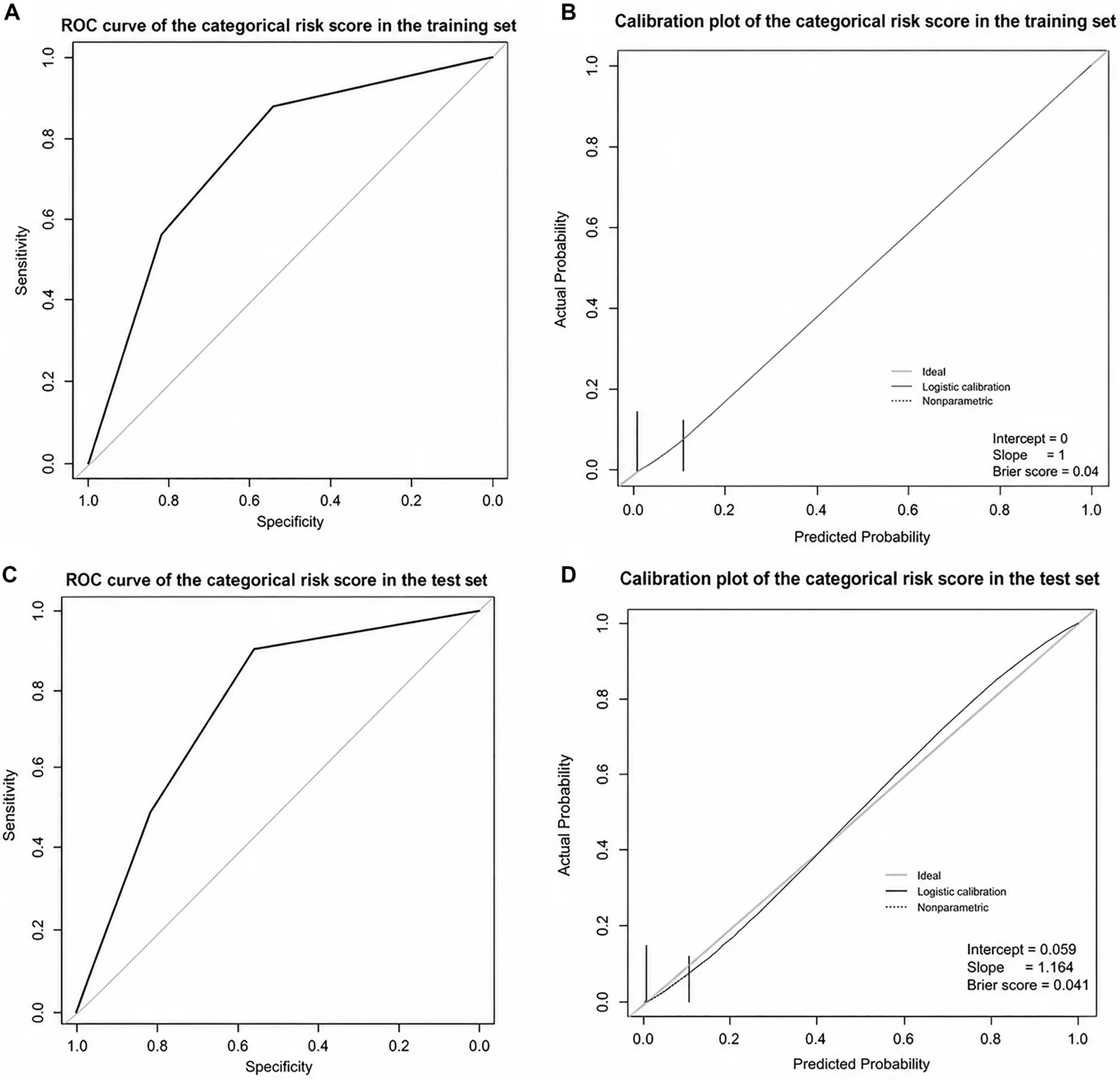
**ROC curves and calibration plots of the categorical cell population data-based risk score for predicting 30-day mortality in the training and test samples of the baseline cohort.** (A) ROC curve of the categorical risk score in the training sample. (B) Calibration plot of the categorical risk score in the training sample, showing excellent agreement between predicted and observed 30-day mortality (intercept = 0, slope = 1.0; Brier score = 0.04). (C) ROC curve of the categorical risk score in the test sample. (D) Calibration plot of the categorical risk score in the test sample, showing acceptable calibration with only minor deviation from the ideal line (intercept = 0.059, slope = 1.164; Brier score = 0.041). The categorical score showed moderate-to-good discriminatory performance, with an area under the ROC curve of 0.75 (95% confidence interval [CI]: 0.70–0.79) in the training sample and 0.77 (95% CI: 0.69–0.82) in the test sample. **Abbreviations**: AUC, area under the receiver operating characteristic curve; CI, confidence interval; ROC, receiver operating characteristic.

DCA demonstrated that the CPD score yielded the highest net benefit across the clinically relevant threshold range of 5%–10%, surpassing all comparator models. Specifically, PSI, CRP-adjusted, and PCT-adjusted models exhibited negative net benefit at 10%, indicating potential net harm relative to a strategy of treating no one. Categorical reclassification analysis showed a statistically significant improvement over CURB-65 (categorical NRI +0.444, 95% CI 0.075–0.813, *P ═* 0.018; continuous NRI +0.645, *P ═* 0.002). This improvement was primarily driven by the correct upclassification of 9 out of 17 cases (53%) initially assigned to low or intermediate risk. The non-significant results for continuous reclassification metrics are attributed to the known instability of these indices in cohorts with a low number of events (*n* ═ 32 events) and do not negate the presence of incremental utility.

### Mortality prediction: baseline and follow-up CPD values as prognostic markers

An additional analysis was conducted on patients with Day 3–5 follow-up, when supplementary blood samples were obtainable. Among patients who survived to the Day 3–5 landmark assessment, 79 subsequently died before Day 30. Patients in this cohort who died were older, exhibited higher CCI and severity scores, and presented significantly elevated levels of PCT. CPD was evaluated in relation to the requirement for respiratory support therapies during hospitalization, including high-flow nasal cannula, non-invasive ventilation, and invasive ventilation. Significant differences were observed in a subset of CPD parameters (Table S4). Table S5 illustrates changes in CPD from baseline to Day 3–5 between survivors and non-survivors. A multivariate analysis identified COVID status, age ≥ 65 years, male gender, baseline NE-Y ≥ 51.63, MO-Y ≥ 130, and changes from baseline to Day 3–5 (NE-Y ≥ 3.76, MO-Y ≤ −12.49) as predictors of 30-day mortality (Table 2). The adjusted categorized risk score developed in the validation sample yielded an AUC of 0.82 (95% CI: 0.76, **0.88).** This risk score was subsequently categorized into three risk groups, demonstrating the highest risk among patients with ≥ 11 points, with an AUC of 0.76 (95% CI: 0.64,0.87) (Table 3). A Brier score of 0.03 indicated favorable calibration measures.

## Discussion

This study offers insights into the utility of CPD parameters for predicting mortality risk in patients hospitalized with CAP. A predictive score incorporating NE-WY ≥ 617.4, MO-Y ≥ 130, male gender, age ≥ 65 years, and positive COVID status demonstrated high predictive accuracy for mortality (AUC 0.81 (95% CI: 0.76,0.85)). Beyond discrimination, both cohorts exhibited robust calibration. The derivation cohort achieved excellent calibration (intercept = 0.00, slope = 1.00, Brier score = 0.04). Minor deviations observed in the held-out validation cohort (intercept = 0.059, slope = 1.164, Brier score = 0.041) are consistent with the expected natural shrinkage of model performance when applied to independent data. Notably, the nearly identical Brier scores across both cohorts further support the model’s stability and generalizability, indicating robust calibration and overall predictive accuracy. Furthermore, analysis of CPD changes from baseline to Day 3–5 revealed that changes in NE-Y ≥ 3.76 and MO-Y ≤ -12.49 were significantly associated with 30-day mortality. External validation in independent populations is warranted to confirm the generalizability and clinical applicability of the proposed cutpoints and point weights.

Early identification of pneumonia patients at risk of poor prognosis is crucial for implementing targeted interventions. In recent decades, various prognostic tools, including severity scores and inflammatory biomarkers, have been evaluated. However, clinicians’ experience in assessing severity remains a cornerstone of clinical management. Novel hematology analyzers have recently been developed to provide CPD, which accurately reflect morphological changes in blood cells in response to infection [8]. This study of white blood cells describes variations in leukocyte morphology quantifiable by CPD numerical values, reflecting the activation status of neutrophils, lymphocytes, and monocytes, thereby providing indirect information on the dysregulated immune response.

Our study demonstrates the utility of these parameters in predicting 30-day mortality risk among hospitalized CAP patients, as they reflect stress response and defense mechanisms against infection. Several authors have described monocyte activation as a response to sepsis [18–20]. Similarly, neutrophil fluorescence intensity and monocyte internal structure have been correlated with sepsis [21]. Our group previously identified MO-X and neutrophil signal fluorescence intensity (NE-SFL)—related to nucleic acid content and internal structure of neutrophils and monocytes—as the most relevant CPD for sepsis prediction, leading to the development of a NEMO score for 30-day mortality with an AUC of 0.932 (0.931–0.934) [10].

A recent study comparing patients with common CAP, severe CAP, and healthy subjects demonstrated that combining white blood cell count (WBC) and Mon-XW, which reflect morphological changes in neutrophils and monocytes, was effective in detecting patients with severe CAP [22]. Interestingly, this model showed superior predictive accuracy compared to CRP and PCT. Similarly, in our study, PCT was significantly higher among patients who died; however, a sub-analysis including PCT revealed a lack of significance. Notably, MO-Y and NE-Y, consistently selected in both baseline and follow-up models, were also significantly associated with the requirement for respiratory support therapies, further supporting their potential clinical relevance. Moreover, the continuous CPD score achieved discrimination comparable to CURB-65 and PSI, the two gold-standard severity scores, while offering a key practical advantage: CPD are derived from routine CBC parameters automatically generated at admission, without additional cost or delay. In the categorical analysis, the CPD score significantly outperformed both CURB-65 and PSI, the scores most commonly used in dichotomized clinical decision-making. Additionally, the CPD score demonstrated superior net clinical benefit at the most actionable decision thresholds and statistically significant reclassification improvement over CURB-65 when evaluated using clinically defined risk categories—the appropriate primary metric in a low-event cohort. Taken together, these findings position the CPD score as a clinically competitive and more accessible alternative for rapid risk stratification in CAP.

Another study investigated patients admitted to the ICU, comparing those with sepsis to those without, utilizing an additional healthy control cohort [15]. The authors highlighted the utility of neutrophil fluorescence intensity and monocyte internal structure for sepsis management, demonstrating a strong correlation with the SOFA score. Our results align with those of Buoro et al., and a model incorporating NE-WY ≥ 617.4 and MO-Y ≥ 130 exhibited high predictive accuracy for mortality in a multicenter study conducted on a larger sample. Focusing on SARS-CoV-2 infection, CPDs have proven useful for COVID-19 diagnosis and prognosis [14, 23]. It has been postulated that data from CPD parameters and concentrations of light immunoglobulin chains can differentiate between severe and non-severe COVID-19 [16, 24].

Despite the current use of severity scores and inflammatory biomarkers, clinicians still face challenges in identifying patients with CAP at higher risk of adverse outcomes. Our study provides evidence on the accuracy of CPD parameters as a prognostic tool. CPD parameters are readily available from routine blood laboratory tests, offering additional evidence of immune response activation that can aid in identifying high-risk patients. Thus, CPD provides clinicians with rapid, accessible, and cost-effective information.

Notably, we were able to obtain blood samples for analysis at an early follow-up and assess changes from baseline to Day 3–5, allowing for detailed monitoring of the inflammatory response. The Day 3–5 model was designed to evaluate the prognostic value of early biomarker kinetics and clinical evolution during the initial days post-admission. Therefore, it should be interpreted as a landmark analysis restricted to patients with available Day 3–5 samples rather than as an admission-based prediction model. Study data indicate that not only baseline CPD parameters but also changes during hospitalization can be valuable in detecting patients at high risk of clinical deterioration.

This study possesses several strengths. Firstly, its prospective and multicenter design; second, a large sample size compared to existing literature; third, the comparison with current systemic inflammation biomarkers; and fourth, the collection of early follow-up changes in a substantial number of patients. However, it also has certain limitations. The analysis necessitates the use of a Sysmex XN analyzer (although this is a widely adopted method, being the European market leader). A further limitation is that approximately 70% of the study population had SARS-CoV-2 infection. Therefore, the performance and generalizability of the proposed score in patients with non-COVID community-acquired pneumonia require further evaluation in more representative cohorts. Additionally, while the CCI was assessed in all patients and is a widely used and validated tool for evaluating comorbidity burden, it has recognized limitations as it does not incorporate functional status, frailty, or baseline dependency [25]. Future studies should integrate functional and frailty assessments to better characterize patient risk. It should be noted that the Day 3–5 analysis was designed as a descriptive and prognostic assessment of CPD kinetics rather than a formal dynamic trajectory model. Approaches such as joint models, latent class trajectory analysis, or mixed-effects models with time-varying covariates would require denser longitudinal sampling across multiple time points and larger event counts than were available in the present dataset. Future prospective studies incorporating serial CPD measurements should prioritize these methodological approaches to better characterize individual response trajectories and their relationship with clinical outcomes. Finally, future research should focus on different etiological severities.

## Conclusion

In conclusion, the analysis of CPD, expressed as quantitative parameters, demonstrated discriminatory performance comparable to established prognostic tools and may represent a straightforward approach for risk stratification in patients hospitalized with CAP.

**LEUCOCAP Study Group:** Ane Uranga, Amaia Artaraz, Aitor Ballaz, Sandra Dorado, Silvia Pascual (Department of Pneumology, University Hospital of Galdakao-Usansolo, Galdakao, Spain), Urko Aguirre, Jose María Quintana, Ane Villanueva (Osakidetza Basque Health Service, Galdakao-Usansolo University Hospital, Research Unit, Galdakao, Spain), María Jose Lopez de Goicoechea (Department of Microbiology, Universitary Hospital of Galdakao-Usansolo, Galdakao, Spain), Carmen Mar, Eloisa Urrechaga, Cristina Ponga (Laboratory, Universitary Hospital of Galdakao-Usansolo, Galdakao, Spain) Ignacio Arriaga, Maider Intxausti, (Department of Pneumology, Universitary Hospital of Basurto, Bilbao, Spain), Domingo Fernandez (Department of Microbiology, Universitary Hospital of basurto, Bilbao, Spain), Itziar Benito (Laboratory, Universitary Hospital of Basurto, Bilbao, Spain), Carlos Ruiz-Martinez, Javier Ugeda (Department of Pneumology, Universitary Hospital of San Pedro Logroño, La Rioja, Spain),Pilar Sanz, Ivan Bernardo (Department of Biomedical Diagnostics, Universitary Hospital of San Pedro Logroño, La Rioja, Spain), Pedro Pablo España (Department of Pneumology, Universitary Hospital of Galdakao-Usansolo, Galdakao, Spain).

## Supplemental data

Supplemental data are available at the following link: https://www.bjbms.org/ojs/index.php/bjbms/article/view/14362/4232.

## Data Availability

Please contact the first author for data requests.
